# Conditions favoring phagotrophy can lead to larger cell sizes in some freshwater mixoplankton

**DOI:** 10.1093/plankt/fbae077

**Published:** 2025-01-30

**Authors:** Frédérick Girard, Amélie Garnier, Riley Hughes, Charlie Sarran, Eric Harvey, Beatrix E Beisner

**Affiliations:** Department of Biological Sciences, University of Quebec at Montréal (UQAM), C.P. 8888 Succ. Centre-Ville, Montréal, QC, H3P 3P8, Canada; Interuniversity Research Group in Limnology/Groupe de Recherche Interuniversitaire en Limnologie (GRIL), CP 6128 Succ. Centre-Ville, Montréal, QC, H3C 3K7, Canada; Department of Biological Sciences, University of Quebec at Montréal (UQAM), C.P. 8888 Succ. Centre-Ville, Montréal, QC, H3P 3P8, Canada; Department of Biological Sciences, University of Quebec at Montréal (UQAM), C.P. 8888 Succ. Centre-Ville, Montréal, QC, H3P 3P8, Canada; Interuniversity Research Group in Limnology/Groupe de Recherche Interuniversitaire en Limnologie (GRIL), CP 6128 Succ. Centre-Ville, Montréal, QC, H3C 3K7, Canada; Interuniversity Research Group in Limnology/Groupe de Recherche Interuniversitaire en Limnologie (GRIL), CP 6128 Succ. Centre-Ville, Montréal, QC, H3C 3K7, Canada; Département de Sciences Biologiques, University of Montréal, CP 6128 Succ. Centre-Ville, Montréal, QC, H3C 3K7, Canada; Interuniversity Research Group in Limnology/Groupe de Recherche Interuniversitaire en Limnologie (GRIL), CP 6128 Succ. Centre-Ville, Montréal, QC, H3C 3K7, Canada; Département des Sciences de l’Environnement, Université of Québec in Trois-Rivières (UQTR), 3351 Blvd des Forges, Trois-Rivières, G8Z 4M3, Canada; Department of Biological Sciences, University of Quebec at Montréal (UQAM), C.P. 8888 Succ. Centre-Ville, Montréal, QC, H3P 3P8, Canada; Interuniversity Research Group in Limnology/Groupe de Recherche Interuniversitaire en Limnologie (GRIL), CP 6128 Succ. Centre-Ville, Montréal, QC, H3C 3K7, Canada

**Keywords:** density, nutrient ratios, carbon, bacterivory, cell size

## Abstract

Cell size is a critical regulator of many metabolic processes in protists. We explored whether body size and abundances vary consistently in phytoplankton capable of both autotrophy and heterotrophy (mixoplankton) by manipulating environmental stoichiometric conditions in a mesocosm experiment. We applied two allochthonous subsidy treatments: high C: nutrient ratios (leaves) should favour bacterivory through phagotrophy, while low ratios (insects) should favour autotrophy. We identified three focal mixoplankton taxa, common in our study system and that represented facultative (*Cryptomonas* sp. and *Plagioselmis* sp) and more obligate phagotrophs (*Ochromonas* sp.). *Ochromonas* was largest in the leaf treatment, which were also associated with larger sizes in *Cryptomonas* (but not the other cryptophyte). The obligately mixotrophic *Ochromonas* responded more significantly to conditions favouring phagotrophy than did the facultative phagotrophic cryptophytes. All mixoplankton taxa densities declined with insect subsidies that favour autotrophy. Future research should examine a wider range of mixoplankton under varying ecological conditions.

## INTRODUCTION

Cell size is a key regulator of unicellular organism resource acquisition. It affects photosynthetic efficiency (Morel and Bricaud, 1981), preferred predator–prey size ratios (Hansen *et al*., 1994) and nutrient absorption. The latter is advantaged by a smaller cell size through surface-to-volume ratios (Grover, 1995; Chakraborty *et al*., 2017), while a larger size yields highest resource encounter rates (Andersen *et al*., 2015). Modelling and empirical studies have demonstrated that mixotrophy may favour larger organism size within plankton food webs (Ward *et al*., 2016; Barton *et al*., 2013). Similarly, Chakraborty *et al*. (2017) demonstrated in their model that larger mixoplankton should be more phagotrophic while smaller mixoplankton should be more phototrophic.

The feeding strategies of mixoplankton can be influenced by the relative ratios of carbon to nutrients in their environment: low ratios favouring autotrophy and high ratios favouring heterotrophy through greater bacterial production (Carney *et al*., 2016). Allochthonous sources to freshwaters include deciduous tree leaves, with high C: nutrient ratios. We tested whether the addition of such poor nutrient subsidies, promoting heterotrophic conditions, leads to larger mixoplankton cell sizes. We compared these with insect larvae allochthonous inputs, comparatively richer in nutrients, thereby favoring mixoplankton autotrophy. We hypothesized (H1) that mixoplankton will be larger in carbon-rich subsidy treatments (leaf), smallest in nutrient-rich subsidy treatments (insects) and intermediate in insect+leaf treatments.

Secondly, we hypothesized (H2) that chrysophytes (*Ochromonas* sp.) would show larger body size increases than cryptophytes (*Plagioselmis* sp. and *Cryptomonas* sp.). Chrysophytes are generally smaller (Torstensson *et al*., 2024) and would thus benefit more from increasing in size when phagotrophic given that more phagotrophic taxa are expected to be larger (Andersen *et al*., 2015; Chakraborty *et al*., 2017). Moreover, chrysophytes regularly use both photosynthesis and phagotrophy (Holen and Boraas, 1995). Cryptophytes are mainly autotrophic, using phagotrophy when necessary (Juan *et al*., 2016). Together with their larger size, we expected cryptophytes to show a reduced propensity to size shifts under conditions favouring phagotrophy.

Finally, in relation to size shifts and population biomass, it is possible that primarily autotrophic mixoplankton may respond to augmented nutrient levels via allochthonous inputs with higher reproductive rates in lieu of larger body sizes (Andersen *et al*., 2015), thereby leading to higher densities (Carney *et al*., 2016). Thus, we hypothesized (H3) that mixoplankton population densities would be largest with insect subsidies and lowest with leaf subsidies.

## MATERIALS AND METHODS

The experiment was done in 450 L opaque polyethylene floating mesocosms on Lake Triton, Station de Biologie des Laurentides, Saint-Hippolyte, Quebec, Canada. Mesocosms (replicated 4×) were filled with mixed unfiltered lake water. The leaf treatment received subsides of dried whole maple leaves (*Acer saccharum*), while the Insect treatment received dried, whole mealworm larvae (*Tenebrio molitor*); an insect+leaf treament received equal amounts of both. Controls received no inputs. Measurement of total organic C and nutrient (total nitrogen (TN) and phosphorus (TP)) content of each subsidy demonstrated that the Insect addition increased the nutrient concentrations in the mesocosms. This led overall to lowest C: nutrient ratios (mol:mol C:TN ratio of 8.9) in the Insect treatment, highest ratios in the leaf treatment (35.8) and intermediate values (15.5) in the insect+leaf treatment, and 26.3 in the control. Three subsidy treatments were crossed with three intensity sub-treatments to explore the effect of frequency of additions, keeping total biomass input equivalent (40 g total): 5 g/week for all 8 weeks, 10 g/week over 4 weeks and 20 g/week over 2 weeks. The effect of intensity was never significant in our analyses, and we thus focus only on the effect of subsidy type.

Phytoplankton were sampled at the end of the 8-week period (Day 51). From (125 mL) whole-water Lugol’s preserved samples, 10 mL aliquots were sedimented. Using an inverted Nikon microscope (400×) and NIS-ELEMENTS software (Nikon, U.S.A.), we identified, enumerated and measured (lengths, widths) the three focal mixoplankton genera, common in our experiment, up to a total of 150 organisms or 100 fields of view. Cell biovolumes were estimated using geometric formulae. Analysis of Variance (ANOVA) was used to explore the main effects of the subsidy treatments on biovolumes and densities. Analyses were done using R (version 4.2.2) with packages *ggpubr*, *dplyr* and *ggplot2*.

## RESULTS

**Fig. 1:**
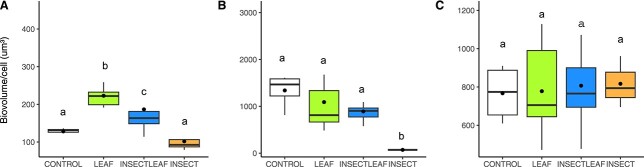
Comparison of cell sizes (biovolume/cell) in the Control and three treatments for: (**A**) *Ochromonas*, (**B**) *Cryptomonas* and (**C**) *Plagioselmis*. Boxes show the data distribution, lines show median values and dots show mean values by treatment. Boxes not sharing a common letter are significantly different according to a Tukey post hoc test.

*Ochromonas* sizes responded to the treatments (F(3,2) = 24.67, *P* < 0.0001) (Fig. 1A), being largest with leaf subsidies (*P* < 0.03). Sizes in the insect treatment were significantly smaller than those in the insect+leaf treatment (*P* < 0.0001). *Cryptomonas* sizes varied significantly between treatments (F(3,2) = 22.3, *P* = 0.0002) (Fig. 1B) with significantly smaller individuals in the insect than leaf (*P* < 0.0001) and insect+leaf (*P* = 0.0002) treatments; these last two did not differ. For *Plagioselmis* no differences in cell sizes were observed (F(3,2) = 0.306, *P* = 0.821) (Fig. 1C).

**Fig. 2:**
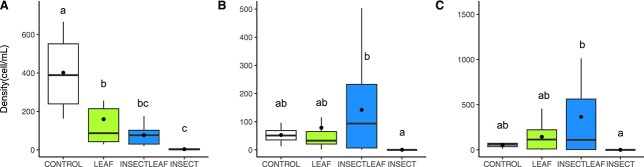
Comparison of cell densities in the Control and three treatments for: (**A**) *Ochromonas*, (**B**) *Cryptomonas* and (**C**) *Plagioselmis*. Boxes show the data distribution, lines show median values and dots show mean values by treatment. Boxes not sharing a common letter are significantly different according to a Tukey post hoc test.

*Ochromonas* densities responded to the treatments (F(2,2) = 9.21, *P* = 0.0002; Fig. 2A), being lower in all treatments than the control (*P* < 0.02). Densities in the leaf were higher than in the Insect treatment (*P* = 0.04) but did not differ from insect+leaf. Density patterns were similar across the two cryptophytes with greatest variation in the insect+leaf treatment and near elimination in the insect treatment. *Cryptomonas* density differences (F(2,2) = 3.19, *P* = 0.04; Fig. 2B) resulted entirely from lower density in the Insect treatment relative to the control (*P* = 0.02). *Plagioselmis* density differences (F(2.2) =3.30, *P* = 0.03; Fig. 2C) resulted from lower values in the Insect relative to the insect+leaf treatment (*P* = 0.03).

## DISCUSSION

We hypothesized (H1) that mixoplankton would be larger in C-rich leaf treatments and smallest in the nutrient-rich Insect treatments. Indeed, the chrysophyte *Ochromonas* fully supported this result. Only partial support was provided by the cryptophytes: only *Cryptomonas* had smallest cell sizes in the nutrient-rich insect treatments. High nutrient ratio inputs thus resulted in smaller mixoplankton cell sizes for two of three taxa, as they presumably focus more on their autotrophic capacities.

Most interestingly however is that the obligate mixoplankton (chryophyte *Ochromonas*) was the only taxon to show significant increases in body size under nutrient limited, C-replete conditions supportive of phagotrophy (leaf additions), as predicted by H2. The lack of response by the more facultative mixoplankton, lends support to observations that cryptophyte phagotrophy is controlled by photosynthetically active radiation (Thurman *et al*., 2012), being generally favoured under extreme environmental conditions (Marshall and Laybourn-Parry, 2002).

Our final hypothesis (H3), predicting increased mixoplankton densities under conditions favouring autotrophy (insects), was not supported; in fact, reduced or equivalent densities were observed relative to the control. *Ochromonas* especially showed reductions with all subsidies. In the case of the leaf treatment reduced densities concur with larger body sizes. In the more nutrient-rich insect subsidies, density declines in the absence of size shifts, suggest that *Ochromonas* were outcompeted by more autotrophic phytoplankton; taxa that were observed to be more common in the Insect-associated treatments (Hughes, 2024).

We cannot completely rule out effects of top-down variation, through differential predation by zooplankton, which may have resulted from our treatments. However, the large shifts in stoichiometry resulting from the different allochthonous input types indicate probable strong bottom-up effects. We encourage further research on size variation in more or less phagotrophic mixoplankton based on our preliminary experiment. Future studies should examine responses across a wider range of taxa and conditions.

## Data Availability

Data and metadata will be made available on the Borealis platform if the manuscript is accepted.
